# Changes in Health Perceptions after Exposure to Human Suffering: Using Discrete Emotions to Understand Underlying Processes

**DOI:** 10.1371/journal.pone.0035854

**Published:** 2012-04-30

**Authors:** Antonia A. Paschali, Efi Mitsopoulou, Valentinos Tsaggarakis, Evangelos C. Karademas

**Affiliations:** 1 Department of Nursing, University of Athens, Athens, Greece; 2 Department of Psychology, University of Crete, Rethymnon, Greece; The University of Melbourne, Australia

## Abstract

**Background:**

The aim of this study was to examine whether exposure to human suffering is associated with negative changes in perceptions about personal health. We further examined the relation of possible health perception changes, to changes in five discrete emotions (i.e., fear, guilt, hostility/anger, and joviality), as a guide to understand the processes underlying health perception changes, provided that each emotion conveys information regarding triggering conditions.

**Methodology/Findings:**

An experimental group (N = 47) was exposed to images of human affliction, whereas a control group (N = 47) was exposed to relaxing images. Participants in the experimental group reported more health anxiety and health value, as well as lower health-related optimism and internal health locus of control, in comparison to participants exposed to relaxing images. They also reported more fear, guilt, hostility and sadness, as well as less joviality. Changes in each health perception were related to changes in particular emotions.

**Conclusion:**

These findings imply that health perceptions are shaped in a constant dialogue with the representations about the broader world. Furthermore, it seems that the core of health perception changes lies in the acceptance that personal well-being is subject to several potential threats, as well as that people cannot fully control many of the factors the determine their own well-being.

## Introduction

Maintenance, improvement or repair of personal health, requires that people engage in an effort to adjust their health-related goals, behaviours, perceptions, and feelings through a process of self-regulation [1]. This process is subject to the influence of several broader personal (e.g., felt symptoms, personality factors) and external factors (e.g., media campaigns) [2]–[6]. One such important external factor is exposure to human suffering, the impact of which on health-related perceptions will be examined in this study.

Exposure to the suffering of other persons, such as watching a disaster or a person in pain is a rather common event of human life [7]. Suffering has been defined by Kleinman [8] as the inter-subjective experience of affliction, which results from either a major aversive event or everyday life circumstances. It usually includes a sense of personal threat [9] and impacts emotions and beliefs about self, goals, and well-being [10]–[12]. It also acts as a reminder of uncertainty in life, uncontrollability, and personal vulnerability [13]–[15].

Previous studies have shown that personal suffering or exposure to the suffering of close persons (e.g., ill family members) can induce changes in health-related perceptions and emotions [16]–[17] and harm personal well-being [18]. Also, exposure to the suffering of others is related to negative changes in perceptions about own health and is coupled with changes in mood [19]. Higher levels of negative mood and lower levels of positive mood were noted after exposure to stimuli of suffering, probably because such a situation induces a sense of threat towards personal goals and status [9]. The mechanisms that underlie health perception change after exposure to stimuli of human suffering are unknown. Nevertheless, changes in discrete emotions can be used as indicators of these mechanisms, provided that within the health-related self-regulation process, perceptions and emotions influence mutually each other.

Different theoretical models emphasize different aspects of the interaction between emotions and perceptions. Certain models place perceptions in the centre of the process (e.g., [20]), others concentrate on the role of emotions (e.g., [21]) while a third category places an equal emphasis on both cognitive and emotional elements (e.g., [22]). Yet, as Consedine [23] underlines, such arguments may in fact be needless since emotions can influence health behaviour through cognitive pathways, while perceptions can also influence health behaviour through emotional channels. Additionally, both perceptions and emotions convey information about the mechanisms that are involved in the health-related self-regulation process [23]–[24]. In this respect, emotions can be used as a guide for understanding cognitive mechanisms and vice versa.

As part of the self-regulation process, emotions are also closely related to personal goals: a positive emotion is likely to occur when goals are achieved, whereas the failure or blockage of goals is associated with the experience of negative emotions [25]. According to Consedine, Magai and Bonanno [26], emotions represent an adaptation that has evolved through the mechanism of natural selection. Each discrete emotion seems to correspond to a specific set of cognitive, behavioural and physiological responses that facilitate the effective adaptation to particular environmental challenges in order to meet basic goals/needs [26]–[28]. For example, fear emerges when there is an immediate danger to personal safety [28]. Thus, emotions convey a significant amount of information regarding the motivational, cognitive, behavioural, and physiological aspects of a reaction. Each emotion refers rather to a class of situations than to particular stimulus [24]. For instance, sadness is similar across different conditions that involve a sense of loss [28].

The aim of this study was to investigate the processes that underlie the impact of exposure to human suffering on health perceptions by using certain discrete emotions as indicators. Our experimental design aimed to examine whether (a) personal health perceptions (i.e., health anxiety, health optimism, health value, and health locus of control) as well as five discrete emotions (i.e., fear, sadness, guilt, anger/hostility, and joy) change as a result of exposure to stimuli of human suffering. These emotions have been related to health outcomes and well-being [23]–[24] and are relevant to the exposure to human suffering, as distressing condition. A further aim (b) was to examine the association between the potential pre/post-experiment changes in emotions and personal health perceptions. This can be used as a guide to increase our understanding of the mechanisms (e.g., cognitive, motivational) that may underlie changes in health perceptions. We presume that each discrete emotion may inform us about the reasons behind personal responses, as well as about the relevant inner processes [24], [26]–[28].

Fear typically arises when a person perceives a threat against physical safety or against important goals and aspects of life, such as well-being [28]–[29]. Fear facilitates avoidance or escape behaviours, as well as information-seeking regarding the source of threat [24]. Also, fear has been related to several health outcomes and health-related behaviour, including heart disease, asthma, more use of cigarettes and alcohol, and overeating [24, for a review]. Sadness has evolved to facilitate adaptation to loss [28]. Loss may refer to a great range of situations, from the failure of a significant goal, to the death of a person, to failure of perceptions about self and the world. Sadness is associated with a focus on inner experience and the self [24], which may result in a more pessimistic evaluation of a situation, and poorer evaluations about physical and psychological well-being [24], [30], [31]. Sadness has been associated with increased risk of myocardial infarction, mortality, and reduced appetite [24, for a review]. It should be noted, however, that the number of studies examining the role of sadness as a discrete emotion in health is very small [24]. Guilt is the product of a real or imagined failure to comply with rules and standards [28]. Feelings of responsibility and an urge to reverse a behaviour or its consequences often accompany guilt [24], [32]. According to a number of studies, guilt can provoke or facilitate the adoption of health-related behaviours, including condom use and exercise [23], [24]. Anger/hostility results when there is a violation of personal rights, perceived injustice, or when a significant goal is blocked [28]. Anger is accompanied with high arousal and strong motivational impulses to ‘rectify injustices’ [26], [28]. Anger has frequently been related to more physical complaints, pain, coronary problems, and early mortality [24]. On the other hand, joy or happiness is the basic positive emotion [33]. There is evidence that increased joy can also increase personal resources, such as self-confidence, outcome expectations and self-efficacy, while it facilitates positive evaluations about self and the world [24], [28]. Joy is also related to conditions of safety and familiarity, as well as to the achievement of important goals [28], [32]. Joy/happiness has been associated with improvement in chronic illness and lower risk of mortality, although there are studies that do not support such associations [24].

Two groups of participants were exposed to a series of either suffering-related images (experimental group) or relaxing stimuli (control group). Emotions and health perceptions were assessed before and after exposure to images so as to detect possible pre/post-experiment differentiations and patterns of associations between emotions and health perceptions changes. It should be noted that relaxing images were preferred for the control group instead of more neutral, in order to test for the possibility that the induction of positive mood can also result in health perception changes. Thus, we would be able to examine whether health perception changes are related to the induction of a mood different from the current.

Our hypotheses were: a) after exposure to stimuli of human suffering participants will report more negative health perceptions and more negative emotions (e.g., more fear and sadness, less joy) in comparison to the control group; b) after exposure to relaxing stimuli (control group) no significant changes in health perceptions are expected, provided that this type of stimuli is assumed to be unrelated to participants' health-related goals and beliefs; c) changes in health perceptions in the experimental group are associated with changes in emotions. Provided that exposure to human suffering can induce a sense of threat towards personal goals [9], we expected that negative changes to health perceptions will be associated with more intense negative emotions (e.g., fear, sadness) and weakened positive emotions (i.e., joy). If fear reflects a perception of threat and personal vulnerability [28], [32] while joy reflects a sense of security and achievement [28], [32], we expected increased fear and decreased joy to be associated with elevated health anxiety and lowered health optimism. We also expected increased sadness and guilt, which are connected to perceptions of personal loss and failure [24], [28], to be correlated with increased health anxiety and decreased optimism, health value and internal health locus of control. Finally, provided that anger/hostility reflects a sense of injustice and of an important goal being at stake [26], [28], we expected anger to be related to more health anxiety, less health value, as well as enhanced internal health locus of control [26].

The hypotheses, regarding the associations between changes in discrete emotions and health perceptions, are only indicative as no previous study has examined such relationships. They are based on the general characteristics of each discrete emotion as previously presented. In any case, we expect that the associations between the pre/post-experiment changes will provide us with information about the processes (motivational, cognitive etc.) that contribute to the modification of each health perception.

## Methods

### Participants

Ninety four healthy undergraduate students (67 females and 27 males; mean age = 20.31 years, SD = 1.84) were randomly assigned in two groups. The first was exposed to images (photos) of human suffering, whereas the second was exposed to relaxing images. Students were recruited through class announcements and participated voluntarily.

### Measures

#### Health-related perceptions

*Health anxiety* was assessed using the corresponding scale from the Multidimensional Health Questionnaire [34]. The scale consists of 5 items (e.g., I feel anxious when I think about my health; Cronbach *a* = .84). Participants responded on a 5-point Likert type scale ranging from 1 (“not at all characteristic of me”) to 5 (“very characteristic of me”). *Health optimism* was assessed using the corresponding scale also from the Multidimensional Health Questionnaire [34]. The scale refers to an expectation that one will continue to experience positive physical health in the future. It consists of 5 items (e.g., I do not expect to suffer health problems in the future; Cronbach *a* = .78). Participants responded on a 5-point Likert type scale ranging from 1 (“not at all characteristic of me”) to 5 (“very characteristic of me”). The Health Value Scale [35] was used to assess the *value attached to health*. It is a four-item scale (e.g., There is nothing more important than good health; Cronbach *a* = .68). Participants responded using a 7-point Likert type scale ranging from 1 (“strongly agree”) to 7 (“strongly disagree”). *Health locus of control* was measured with the Multidimensional Health Locus of Control Scale [36]. The scale consists of 18 items and provides measures of three dimensions of health locus of control: internal (6 items, e.g., I am in control of my health; Cronbach *a* = .69), chance (6 items, e.g., My good health is largely a matter of good fortune; Cronbach *a* = .71), and powerful other (6 items, e.g., Health professionals control my health; Cronbach *a* = .60). Participants responded on a 6-point Likert type scale ranging from 1 (“strongly disagree”) to 6 (“strongly agree”).

#### Emotions

Specific emotions were assessed with the Positive and Negative Affectivity Schedule – Expanded Form (PANAS-X) [37]. PANAS-X consists of sixty items – adjectives measuring eleven specific emotions. For the purposes of this study, we focused on five of them: fear (6 items; e.g., afraid, frightened; Cronbach's *a* = .72); sadness (5 items; e.g., sad, downhearted; Cronbach's *a* = .85); guilt (6 items; e.g., ashamed, guilty; Cronbach's *a* = .80); hostility (6 items; e.g., angry, irritable; Cronbach's *a* = .68); joviality, which was used as a measure of joy/happiness (8 items; e.g., happy, joyful, excited; Cronbach's *a* = .86). Participants responded on a 5-point Likert type scale ranging from 1 (“very slightly or not at all”) to 5 (“extremely”).

### Procedure

The experiment was ‘advertised’ in class as involving reactions to certain visual stimuli. At the day of the experiment ninety-six (96) students were given a set of questionnaires (i.e., health value, health anxiety, health optimism, health locus of control, and emotions). They were instructed to complete the questionnaires having in mind their present condition. They were also asked about their current and recent health status. Two students who reported a recent or current health problem were excluded from the procedure. Thus, 94 participants were randomly assigned to the experimental or the control group. Participants were seated, in groups of about 15 persons per time, in a quite square room, at separate desks behind a data projector that projected against the wall. When seated, participants were instructed to relax and stay quiet with their eyes shut for about 2 minutes. After that period, the presentation commenced. Each image was projected for 10 seconds. A series of 25 photos were presented to each group.

The experimental group was exposed to photos of natural and manmade disasters, ruin and grief, whereas the control group was exposed to a set of relaxing images. As detailed in Karademas [19], the images (photos) were derived from a larger pool of photos coming from the official websites of major broadcasting networks. This initial pool of photos was originally displayed to a small group of post-graduate students, who were asked to rate the degree to which each image was “representative of human suffering; that is situations which provoke great pain and distress to those involved”. The 25 images with the higher mean rating were included in the experiment. Using the same procedure, from an equal initial pool of photos presenting various relaxing situations, the 25 with the higher mean rating were included in the experiment. The set of suffering-related images consisted of 5 photos showing accidents (e.g., motor accident victims), 9 photos showing violence-involving situations (e.g., war acts), 4 photos of natural disasters, 4 of human miserability (e.g., famine) and 3 photos of grieving reactions. The set of relaxing images consisted of photos showing people in relaxing (10 photos; e.g., walking by the beach; playing with animals) or sportive activities (2 photos; e.g., playing football), 5 photos of parents playing with their children, 4 photos of calming down activities (e.g., swimming), and 4 beautiful sceneries.

Participants in both groups were instructed to carefully watch and reflect on the projected images. No other instruction was provided. At the end of the presentation, participants were asked to complete the initial set of questionnaires having in mind their condition at that particular moment. After the completion of the experiment, participants received information about its nature, the purpose and the procedures to exclude the possibility of a longer negative impact (especially due to exposure to suffering-related images).

### Ethics Statement

The study was conducted in accordance with the ethical standards adopted by the European Federation of Psychologists' Association (available in http://www.efpa.be/ethics.php), and was approved by the Ethics Committee of the Department of Psychology, University of Crete.

## Results

A one-way multivariate analysis of variance (MANOVA) across all variables assessed before the experiment (i.e., health anxiety, health optimism, health locus of control, health value, and emotions) with gender as the independent variable was performed. No significant differences were observed [Wilks *λ* = .90; *F*(11, 82) = .84, *p*>.10]. Thus, all subsequent analyses were pooled over gender. The means and standard deviations of all variables, before and after the experiment, are presented in Table 1.

**Table 1:** Personal Health Perceptions and the Specific Emotions Before and After the Experiment.

	Experimental group		Control group	
	Before	After	Before	After
Health anxiety	13.85 (4.99)	16.68 (3.88)	14.21 (4.79)	13.42 (4.99)
Health optimism	17.79 (4.27)	15.19 (4.39)	16.47 (4.18)	16.32 (4.33)
Health value	22.57 (5.27)	24.79 (3.16)	22.08 (5.69)	22.06 (5.41)
HLoC – Internal	25.79 (4.36)	23.51 (4.66)	25.81 (3.86)	25.85 (4.11)
HLoC – Chance	18.66 (6.87)	19.22 (6.20)	18.49 (5.87)	18.45 (6.34)
HLoC – Significant others	21.11 (6.09)	20.81 (5.23)	19.43 (5.07)	20.53 (5.31)
Fear	7.72 (2.05)	10.87 (4.78)	8.11 (6.78)	8.68 (3.34)
Hostility	8.79 (4.12)	11.00 (4.43)	8.66 (3.30)	9.19 (3.81)
Guilt	8.06 (2.73)	10.38 (5.08)	8.36 (3.74)	8.15 (3.09)
Sadness	8.34 (4.18)	10.66 (3.84)	8.28 (4.19)	8.25 (3.45)
Joviality	24.08 (6.88)	19.43 (6.96)	24.00 (6.60)	25.36 (8.42)

Means and Standard Deviations (in Parentheses).

*Note.* HLoC = Health locus of control.

We examined the differences between groups before and after the experimental procedure, a 2 (time)×2 (group) repeated measures MANOVA was performed. According to the results, a significant time×group effect was detected [Wilks *λ* = .44; *F*(11, 82) = 9.45, *p*<.001; partial *η* ^2^ = .56]. The time (pre/post experiment) effect was also statistically significant [Wilks *λ* = .52; *F*(11, 82) = 6.84, *p*<.001; partial *η* ^2^ = .48], but not the group effect [Wilks *λ* = .87; *F*(11, 82) = 1.10, *p*>.10; partial *η* ^2^ = .12].

**Figure 1:**
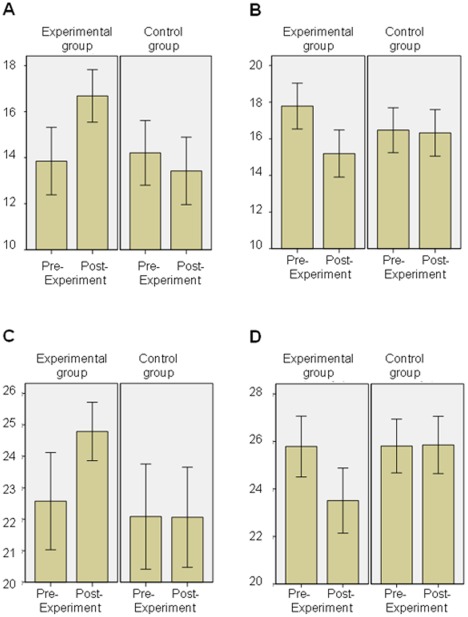
Health anxiety, health optimism, health value, and internal health locus of control before and after the exposure to images. Health anxiety (A) health value (C) were significantly increased after participants were exposed to images of suffering (experimental group) while these measures were unaffected for the participants of the control group. On the contrary, health optimism (B) and internal health locus of control (D) were decreased in the experimental group after the experiment while the control group remained unaffected. Error bars represent +/− 95% CI.

The univariate ANOVAs revealed significant differences before and after the experimental intervention regarding health anxiety [*F*(1, 92) = 15.17, *p*<.001; partial *η* ^2^ = .14], health optimism [*F*(1, 92) = 22.46, *p*<.001; partial *η* ^2^ = .20], internal health locus of control [*F*(1, 92) = 10.84, *p*<.005; partial *η* ^2^ = .11], and health value [*F*(1, 92) = 8.31, *p*<.01; partial *η* ^2^ = .08] (see Figure 1). Also, significant differences were found in all emotions: fear [*F*(1, 92) = 17.39, *p*<.001; partial *η* ^2^ = .08], hostility [*F*(1, 92) = 9.38, *p*<.005; partial *η* ^2^ = .09], guilt [*F*(1, 92) = 7.01, *p*<.01; partial *η* ^2^ = .07], sadness [*F*(1, 92) = 10.04, *p*<.005; partial *η* ^2^ = .10], and joviality [*F*(1, 92) = 5.23, *p*<.05; partial *η* ^2^ = .05]. Regarding the time×group interaction, significant effects were found for anxiety [*F*(1, 92) = 45.58, *p*<.001; partial *η* ^2^ = .34], health optimism [*F*(1, 92) = 17.85, *p*<.001; partial *η* ^2^ = .16], internal health locus of control [*F*(1, 92) = 11.68, *p*<.005; partial *η* ^2^ = .12], health value [*F*(1, 92) = 8.64, *p*<.005; partial *η* ^2^ = .09], as well as fear [*F*(1, 92) = 8.31, *p*<.01; partial *η* ^2^ = .08], hostility [*F*(1, 92) = 4.52, *p*<.05; partial *η* ^2^ = .05], guilt [*F*(1, 92) = 10.12, *p*<.005; partial *η* ^2^ = .10], sadness [*F*(1, 92) = 10.41, *p*<.005; partial *η* ^2^ = .10], and joviality [*F*(1, 92) = 19.28, *p*<.001; partial *η* ^2^ = .17]. No other significant effects were found.

In order to investigate specific mean differences, the individual *t*-tests as provided by the repeated measures MANOVA, were examined. To prevent alpha inflation at this level of analysis, a Bonferroni correction for multiple comparisons was applied. Also, the 95% confidence intervals were computed so as to provide further insight into plausible mean differences. Table 2 summarizes the significant findings. There were no group differences before the experiment regarding the variables examined in the study. On the contrary, after the experiment, those exposed to images of human suffering reported higher levels of health anxiety and value attached to health, less internal health locus of control, as well as more fear, hostility, guilt, sadness, and less joviality, in comparison to the control group. The post-experiment difference between the two groups, as far as health optimism is concerned, was not statistically significant. This indicates that the significant time×group effect on optimism corresponds only to pre/post-experiment changes within the experimental group.

**Table 2:** The Significant Mean Differences Among the Experimental and the Control Group.

	Variable	Mean difference	*t*-test	95% Confidence Intervals		*η* ^2^
				Lower	Upper	
Pre-experiment	Health anxiety	−.36	−.36	−2.37	1.64	.001
	Health optimism	1.32	1.51	−.41	3.05	.02
	Internal HLoC	−.02	−.25	−1.71	1.67	.001
	Health value	.49	.43	−1.76	2.74	.002
	Fear	−.39	−.62	−1.61	.84	.004
	Hostility	.13	.17	−1.40	1.66	.001
	Guilt	−.30	−.44	−1.64	1.04	.002
	Sadness	.06	.07	−1.65	1.78	.001
	Joviality	.08	.06	−2.68	2.85	.001
Post-experiment	Health anxiety	3.26	3.53***	1.42	5.09	.12
	Health optimism	−1.13	1.25	−2.91	.66	.02
	Internal HLoC	−2.34	−2.58**	−4.14	−.54	.07
	Health value	2.73	2.98**	.91	4.54	.09
	Fear	2.19	2.58*	.50	3.88	.07
	Hostility	1.81	2.12*	.12	3.50	.05
	Guilt	2.23	2.58*	.51	3.96	.07
	Sadness	2.41	3.20**	.91	3.90	.10
	Joviality	−5.93	−3.45**	−8.82	−2.38	.12

The mean differences among the experimental and the control group before and after the experiment as derived from the repeated measured MANOVA, the corresponding t-tests and confidence intervals.

*Note.* HLoC = Health locus of control.

* *p*<.05, ** *p*<.01, *** *p*<.001.

The correlations between health perceptions and emotions for the entire sample before the experiment are presented in Table 3. According to these results, with the exception of just one case, no significant relations between emotions and health perceptions were found. In order to determine whether changes in health value, health anxiety, health optimism and internal health locus of control were associated with changes in emotions, we subtracted the pre-experiment from the post-experiment score in each health perception and emotion. Then, we examined their correlations for the experimental and the control group.

**Table 3:** Descriptive Statistics and Intercorrelations of Health Perceptions and Emotions.

Variable	1	2	3	4	5	6	7	8	9	10	11
1. Health anxiety	1.00										
2. Health optimism	−.15	1.00									
3. HLoC – Internal	−.07	−.01	1.00								
4. HLoC – Chance	.19†	−.03	−.33**	1.00							
5. HLoC – Others	.03	.11	.28**	−.36**	1.00						
6. Health value	.07	.19†	.29**	−.34**	.26*	1.00					
7. Fear	−.01	−.11	−.17	.11	.15	−.12	1.00				
8. Hostility	.05	−.08	−.19	.13	−.02	−.16	.55**	1.00			
9. Guilt	−.06	−.08	−.01	.08	.20*	−.10	.75**	.50**	1.00		
10. Sadness	−.01	−.11	−.12	.12	.15	−.23*	.71**	.57**	.84**	1.00	
11. Joviality	−.01	−.02	.09	−.03	−.08	−.10	−.22*	−.21*	−.37**	−.39**	1.00
Mean (*N* = 94)	14.03	17.13	25.80	18.57	20.27	22.33	7.91	8.72	8.21	8.31	24.04
SD (*N* = 94)	4.86	4.25	4.09	6.35	5.64	5.46	2.97	3.71	3.26	4.16	6.71

Descriptive Statistics and Intercorrelations of Health Perceptions and Emotions for the Entire Sample Before the Experiment (N = 94).

*Note.* SD = Standard deviation. HLoC = Health locus of control.

† *p*<.10, * *p*<.05, ** *p*<.01.

As presented in Table 4, health anxiety showed statistically significant associations with four emotions: guilt, hostility, and sadness showed a positive association, while joviality showed a negative association. Health optimism was related only to guilt in a negative way. Internal health locus of control was tentatively correlated to sadness in a negative way. As far as the control group is concerned, no significant associations between changes in emotions and health perceptions were noticed.

**Table 4:** Post-experiment Correlations (Pearson's r) of Health Perceptions Changes to Discrete Emotions Changes for the Experimental and the Control Group.

	Fear	Hostility	Guilt	Sadness	Joviality
**Experimental group**					
Health anxiety	.17	.29*	.34*	.42**	−.42**
Health optimism	−.11	−.14	−.30*	−.13	.19
Internal locus of****control	.02	−.01	−.10	−.28†	.02
Health value	.04	−.32*	.02	−.17	.24
**Control group**					
Health anxiety	.03	.10	−.14	.07	−.07
Health optimism	−.03	.01	−.03	.12	−.15
Internal locus of****control	−.01	−.03	−.02	.04	−.16
Health value	.04	−.02	.18	−.06	.17

The Post-experiment Correlations (Pearson's r) of Health Perceptions Changes to Discrete Emotions Changes (Pre-Post Experiment) for Each Group.

† *p* = .06, * *p*<.05, ** *p*<.01.

## Discussion

In a recent experimental study [19], exposure to stimuli of human suffering was found to be associated to changes in personal health perceptions, as well as in mood. Drawing on these findings, the purpose of the present study was to examine the relationships between possible changes in personal health perceptions and changes in five discrete emotions after exposure to stimuli of human suffering. Within a self-regulation perspective, according to which perceptions and emotions are closely related to each other [22], [23], the latter were used as a guide to understand the processes underlying health perception changes [26]–[28].

Overall, the findings provided support to our hypotheses. Exposure to stimuli of human suffering had an impact on health perceptions and emotions. Participants exposed to these stimuli reported more health anxiety and value attached to health after the experiment, as well as lower health-related optimism and internal health locus of control, in comparison to a control group of participants exposed to relaxing images. They also reported more fear, guilt, hostility and sadness, and less joviality. As suggested by Leventhal et al. [22], crucial information can cause changes in the cognitive and emotional representations of personal health. Exposure to stimuli of human suffering seems to act as such an important source of information that caused changes in personal health perceptions and also emotional arousal.

According to the findings, the differences between the experimental and the control groups had rather to do with post-exposure changes within the first group, as no significant changes in health perception or the emotional state were noticed in the control group. This finding underlines that health perception changes are not the ‘automatic’ or self-evident result of exposure to mood-inducing situations (that is negative changes in the case of distressing stimuli and positive changes in the case of relaxing stimuli), but are connected to the specific content of these stimuli.

Changes in health perceptions after exposure to stimuli of human suffering were related to changes in emotions in the experimental group, whereas no such relations were found in the control group. In particular, increased health anxiety was related to higher levels of hostility, guilt and sadness, as well as lower levels of joviality. Likewise, weakened health optimism and health value were related to increased levels of guilt and hostility, respectively. In other words, it seems that changes in each of the above mentioned health perceptions were related to changes in particular emotions. Provided that each discrete emotion corresponds to a specific set of motivational, cognitive, behavioural and physiological responses [24], [26]–[28], the differentiation in the pattern of associations between health perceptions and emotions, before and after the experiment, can be used as a guide to understand the processes that were triggered after exposure to human suffering and probably underlie changes in health perceptions.

The fact that health anxiety was unrelated to any emotion before the experiment (or in the control group after the experiment) indicates that it was exposure to stimuli of human suffering that initiated an array of inner responses that resulted in increased concern about personal health. Changes in emotions can give us insight into this process: increased anger/hostility probably reflects the realization of an “unjust” world [28]; increased sadness probably reflects a failure in perceptions about (a capable) self and/or (a predictable) world [24], [28], while increased guilt possibly indicates a sense of responsibility for a failure [24]; decreased joviality possibly indicates a perception of lost safety [28], [32]. All these, as a result of exposure to human suffering, seem able to induce a sense of personal vulnerability and thus increase concern about one's own health. It is puzzling why this feeling of personal vulnerability was not related to fear. A possible explanation might be that fear corresponds to a possibility of a future risk [28], [29], whereas exposure to human suffering acted as a reminder of the actual personal vulnerability that is already present, although ‘invisible’ [13]–[15].

Similarly, the association of guilt to health optimism indicates that those exposed to stimuli of human suffering felt a real or imagined failure to comply with standards [24] that probably refer to well-being. In other words, exposure to suffering made them ponder the actual fragility of well-being and perhaps their own failures or inabilities to preserve it. Thus, they restrained the expectations about personal health in the future. Regarding the negative association between health value and hostility, and provided that anger/hostility arises when an important goal is perceived as unduly blocked [26], [28], it is possible that health value was diminished as a reaction to the realization that another important goal/need is endangered. That is, it is possible that exposure to human suffering also acted as a reminder of the actual threats against the general (besides the personal) welfare, which represents a very important goal for humans [38]. This in turn resulted in questioning and diminishing the value originally attached to personal health. Finally, as far as the internal health locus of control is concerned, no statistically significant correlation to emotions were found. Nevertheless, given that a marginally significant correlation to sadness was detected, a tentative explanation of the decreased internal locus of control might be that the loss of perceived safety enabled participants to consider their personal inability to exert full personal control over own health.

In conclusion, the findings of this study showed that perceptions about personal health can be affected not only by own experiences, but also by the exposure to the experience of unrelated or distant people. It should not escape attention that environment is a primary source of learning for humans [39]. It seems that human beings live within a “cognitively-emotionally connected word” where perceptions about self are related to perceptions about the others and the world. In this context, exposure to human suffering was found to act as a fuse against pre-exposure perceptions of personal health, through the activation of certain cognitive-emotional mechanisms. To the extent that each emotion conveys information regarding triggering conditions [26]–[28], it appears that in the core of health perception changes lies the acceptance that personal well-being is subject to several potential losses, as well as that people cannot fully control several factors that are important for health and well-being. This recognition probably contributed to the removal of optimistic biases and, through this, to the modification of health perceptions.

The findings of this study should be considered in relation to certain limitations. First, no re-assessment or follow-up took place so as to examine whether and for how long the changes in health perceptions are maintained after exposure to stimuli of human suffering. Second, in this experimental study we did not examine the role of factors of primary importance with respect to the impact of distressing stimuli on health perceptions and emotions, such as personality or personal history. The stimuli used in the experiment were referring to situations rather uncommon to the participants' current and previous experiences. Finally, participants were young students; older persons probably perceive themselves and the world in a different way. Nevertheless, we believe that the understanding of the impact of exposure to suffering on everyday functioning, the underlying mechanisms, as well as the use of discrete emotions as a guide to understand these mechanisms, are issues that deserve extensive investigation as they represent significant aspects of the human behavior.
