# Correction: Changes in Health Perceptions after Exposure to Human Suffering: Using Discrete Emotions to Understand Underlying Processes

**DOI:** 10.1371/annotation/ab7ad045-2596-4278-8506-dc9ba2bdbcb2

**Published:** 2012-07-09

**Authors:** Antonia A. Paschali, Efi Mitsopoulou, Valentinos Tsaggarakis, Evangelos C. Karademas

There was an error in affiliation 1 for author Antonia A. Paschali. Affiliation 1 should be: Department of Mental Health and Behavioral Sciences, Faculty of Nursing, University of Athens, Athens, Greece 

